# Microbial diversity and peptide dynamics during the spontaneous fermentation of whey from the Yunnan ethnic cheese Rushan

**DOI:** 10.1016/j.fochx.2026.104464

**Published:** 2026-09-21

**Authors:** Rong Tang, Huimin Li, Faguang Yang, Xing Wan, Xingying Dai, Junfei Chen, Chenrui Zhang, Ling Zou, Qiao Shi

**Affiliations:** a Agro-Product Processing Research Institute, Yunnan Academy of Agricultural Sciences, Kunming 650221, China; b Yunnan Ouya Dairy products Co. Ltd, Dali 671000, China; c Department of Microbiology, Faculty of Agriculture and Forestry, University of Helsinki, Helsinki 00790, Finland; d Institute of Flowers, Yunnan Academy of Agricultural Sciences, 650000, China

**Keywords:** Rushan whey, Spontaneous fermentation, Microbial diversity, Peptidomics, Bioactive peptides

## Abstract

Rushan whey (RW) is a byproduct of Rushan cheese production, yet its potential as a source of bioactive peptides remains unexplored. Here, microbial dynamics and peptide profiles were monitored throughout spontaneous RW fermentation. Bacterial diversity declined drastically within the first week, by the end of which *Lactobacillus* (specifically L. *delbrueckii* and *L. helveticus*), dominated the community, followed by *Acetobacter* (specifically *A. fabarum* and *A. pasteurianus*)*.* Fungi, particularly *Saccharomyces cerevisiae* and *Pichia kudriavzevii*, appeared only on day 14. Peptide diversity decreased over time, while β-casein-derived peptides consistently dominated the peptidome. Abundances of total and putative bioactive peptides peaked on days 11 and 7, respectively, before declining markedly thereafter. Variations in total, casein-fraction-derived, and putative bioactive peptides were correlated with specific bacterial taxa. This study is the first to apply peptidomics to elucidate peptide generation dynamics during RW fermentation, providing a scientific basis for RW valorization through targeted, microbe-driven bioactive peptide enrichment.

## Highlights

• Microbial diversity and peptidomics were characterized in Rushan cheese whey.
• Bacterial diversity declined sharply, while fungi emerged late in fermentation.
• Peptide diversity declined, with β-casein peptides dominating the peptidome.
• Total peptides and putative bioactive peptides peaked at days 11 and 7, respectively.
• Specific bacteria were associated with putative bioactive peptide generation.

## 1 Introduction

Rushan is a traditional fermented cheese produced by the Bai ethnic group in Dali, Yunnan Province, China. Unlike industrial cheeses, Rushan is manufactured without using any commercial starter cultures. Instead, the Rushan whey (RW) obtained from the previous batch of production is employed as a natural coagulant following fermentation. The resulting Rushan curd is subjected to heat treatment, stretching, and drying to form the final product (Chen et al., 2021). RW, a by-product of Rushan cheese production, accounts for up to 90% of the initial milk volume. It consists of lactose, caseins, whey proteins, peptides, amino acids, minerals, and water-soluble vitamins, thereby providing a favorable environment for microbial growth and metabolism (Liao, 2021). However, this large volume of whey is often regarded as a low-value waste, and its disposal poses a significant environmental burden due to its high organic load.

The microbial composition of cheese whey varies considerably depending on factors such as geographical origin and processing conditions. For instance, *Lactobacillus delbrueckii*, *Lactobacillus helveticus*, and *Streptococcus thermophilus* were found to be predominant in whey used for the production of Caciocavallo Silano cheese (Ercolini et al., 2008). Meanwhile, *Streptococcus thermophilus* and thermophilic lactobacilli dominated the whey used in PDO Comté cheese production (Lutin et al., 2024). Similarly, in the analysis of residual RW, *Lactobacillus kefiranofaciens* and L. *helveticus* were identified as the predominant microbes (Jiang et al., 2021). Despite these findings, systematic studies on the microbial dynamics and peptidomic profiles of RW during spontaneous fermentation are still lacking.

During the whey fermentation process, microorganisms degrade proteins into peptides. Peptides are short chains of amino acids that are linked by peptide bonds. Certain peptides show bioactivities, including immunomodulatory, antimicrobial, antioxidant, antithrombotic, cholesterol-lowering, hypoglycemic, and antihypertensive effects, with some also exhibiting multifunctionality (Gobbetti et al., 2002). These peptides play a crucial role in the development of sensory attributes in dairy products and contribute significantly to their nutritional value. Consequently, they are of considerable interest in the fields of food science and nutrition (Sánchez-Rivera et al., 2014; Zhang et al., 2023). Milk proteins, particularly caseins and whey proteins, are considered excellent precursors of bioactive peptides because of their high abundance and well-characterized amino acid sequences. Bioactive peptides can be released from these precursor proteins through microbial fermentation, enzymatic hydrolysis, or gastrointestinal digestion.

Rushan production is currently transitioning toward large-scale industrial manufacturing. However, the efficient utilization of RW and the production of RW-derived functional ingredients remain underdeveloped. RW is known to serve as an ideal culture medium for microorganisms. The microbial metabolism in RW enables proteolysis and the release of bioactive peptides, potentially making it a valuable source of bioactive peptides. Previously, Ali et al. discovered antiviral peptides derived from whey proteins fermented by *Limosilactobacillus reuteri* and *Lactobacillus acidophilus* (Ali et al., 2019). Similarly, Shanmugam et al. isolated and characterized antioxidant peptides from enzymatically hydrolyzed buffalo casein (Shanmugam et al., 2015). However, the valorization of RW is hindered by a lack of systematic knowledge concerning peptide composition, precursor protein sources, generation dynamics, and their interplay with the microbiome in this complex matrix. Peptidomics has emerged as a powerful analytical tool that is widely used to examine the composition, structure, function, and dynamic changes of peptides following protein processing, modification, or digestion (Zaloga & Siddiqui, 2004). Although some progress has been made in understanding the dynamics of microbial communities in RW and identifying certain peptide segments (Wei et al., 2024), research on RW peptidomics remains limited.

In the present study, we first analyzed microbial diversity and peptidomic profiles at different stages of spontaneous RW fermentation. Further, we investigated the potential correlations among microbial diversity and peptide profiles. To our knowledge, this study is the first to systematically explore the dynamics of indigenous microorganisms and peptide profiles during the spontaneous fermentation of RW. The findings of this study could provide a scientific foundation for isolating targeted functional strains and designing microbial-driven fermentation strategies to enrich bioactive peptides, thereby facilitating the valorization of RW as an underutilized dairy byproduct.

## 2 Materials and methods

### 2.1 Preparation and collection of samples

RW samples were collected from Yunnan Ouya Dairy Co., Ltd. (Yunnan, China). The whey separated from the Rushan cheese curd was collected and allowed to stand still for subsequent processing. The residual liquid that remained after removing the sediment and surface fat was used as RW. A single batch of RW was subjected to spontaneous fermentation at room temperature for 14 days. Aliquots were sampled in triplicate on days 0, 7, 11, and 14 of fermentation (F0d, F7d, F11d, and F14d). Following collection, the samples were transported to the laboratory on dry ice and stored at −80 °C for further analysis.

### 2.2 Analysis of pH and viable microbial counts in RW

The pH of RW samples was determined using a SevenGo™ pH meter (Mettler Toledo, Greifensee, Switzerland). For the enumeration of viable lactic acid bacteria (LAB) and yeast, 100 μL of RW was spread onto de Man-Rogosa-Sharpe (MRS) agar (Hopebio, China) and yeast extra-peptone-dextrose (YPD) agar (Hopebio, China), respectively. The plates were incubated at 37 °C and 30 °C before enumeration, respectively.

### 2.3 Microbial diversity analysis

Total DNA was extracted using the TGuide S96 magnetic bead DNA extraction kit (Tiangen, Beijing, China) according to the manufacturer's protocol. DNA quality, purity, and concentration were assessed prior to the PCR amplification of the bacterial 16S rRNA gene using universal primers: 27F (5’-AGRGTTTGATYNTGGCTCAG-3′) and 1492R (5’-TASGGHTACCTTGTTASGACTT-3′). The PCR cycling conditions consisted of an initial denaturation at 95 °C for 2 min, followed by 30 cycles of denaturation at 98 °C for 10 s, annealing at 55 °C for 30 s, and extension at 72 °C for 90 s, with a final extension at 72 °C for 7 min.

For fungal community analysis, the internal transcribed spacer (ITS) region was amplified by PCR using the primers ITS1F (5’-CTTGGTCATTTAGAGGAAGTAA-3′) and LR3 (5’-CCGTGTTTCAAGACGGG-3′). The PCR cycling conditions consisted of an initial denaturation at 98 °C for 5 min, followed by 28–30 cycles of denaturation at 98 °C for 20 s, annealing at 54 °C for 30 s, extension at 72 °C for 35 s, and a final extension at 72 °C for 5 min.

The final PCR products were recovered through 1.8% agarose gel electrophoresis and purified using Agencourt AMPure XP Beads. The DNA concentration was measured using the Qubit dsDNA HS Assay Kit. Post quantification, equal amounts of the DNA were pooled, and SMRTbell libraries were constructed using the SMRTbell Express Template Prep Kit 2.0 for sequencing. PCR amplification, product pooling, purification, library preparation, and sequencing were all carried out by Biomek Biotechnology Co., Ltd. (Beijing, China). Negative controls (blank controls without templates) were included during both the DNA extraction and PCR amplification steps. No amplification products were detected in these negative control samples.

Raw sequencing data were initially filtered using Trimmomatic (version 0.33) to remove low-quality bases. The primer sequences were removed using Cutadapt (version 1.9.1). The paired-end reads obtained from previous steps were assembled through USEARCH (version 10), followed by chimera detection and removal using UCHIME (version 8.1). Raw subreads were processed to generate circular consensus sequencing (CCS) reads using SMRT Link (version 8.0). To identify the CCS reads of different samples based on barcode sequences and thus obtain high-quality CCS sequences, lima (v1.7.0) was employed.

Amplicon sequence variant (ASV) analysis was performed using the Divisive Amplicon Denoising Algorithm 2 (DADA2) method implemented in QIIME2 (version 2020.06), and a threshold of 0.005% was applied for ASV filtration.

### 2.4 Extraction of peptides from whey

Peptides were extracted following a previously reported method (Sánchez-Rivera et al., 2014). The samples were transferred into 10 kDa ultrafiltration tubes (Merck KGaA, Germany) and centrifuged at 12,000 ×*g* and 4 °C for 10 min. Peptide fractions with a molecular weight smaller than 10 kDa were collected. A dithiothreitol solution was added to a 20 μg sample (final concentration of 10 mmol/L), followed by reduction at 56 °C for 1 h. Subsequently, an iodoacetamide solution was added (final concentration of 50 mmol/L), and the alkylation reaction was carried out in the dark for 40 min. These reduction and alkylation steps were implemented to prevent artifactual peptide aggregation caused by the spontaneous oxidation of free cysteine residues, improve tandem mass spectrometry (MS/MS) fragmentation efficiency by yielding linear peptide structures, and enhance the detection sensitivity of cysteine-containing peptides through carbamidomethylation (+57 Da), which also facilitates database searching as a fixed modification.

For desalting, the desalting column was first activated with 300 μL of acetonitrile (ACN) and then centrifuged at 3000 ×*g* for 2 min; this step was repeated once again. The column was equilibrated twice with 250 μL of 0.1% trifluoroacetic acid (TFA) via centrifugation at 3000 ×*g* for 2 min. The sample was loaded onto the equilibrated column via centrifugation under the same conditions, and the flow-through was reloaded once to ensure sample binding. The column was washed twice with 250 μL of 0.1% TFA under the same centrifugation conditions to remove salts. Peptides were eluted using 300 μL of 80% ACN/0.1% TFA via centrifugation at 3000 ×*g* for 2 min. The eluate was further desalted and vacuum-dried at 45 °C. Finally, the dried sample was reconstituted in 0.1% formic acid (FA) and centrifuged at 13,000 ×*g* for 15 min; the resulting supernatant was collected for mass spectrometry (MS) analysis.

### 2.5 Peptidomic analysis using liquid chromatography-tandem mass spectrometry (LC-MS/MS)

The reconstituted peptides were separated using an Liquid Chromatography (LC) column (100 μm × 180 mm) packed with Reprosil-Pur 120 C18-AQ (3 μm). Mobile phase A consisted of 0.1% FA, while mobile phase B consisted of 0.1% FA and 80% ACN. The flow rate was set at 600 nL/min, over a total analysis time of 131 min. Gradient elution started with 4% B, which was increased to 40% B over 2 h, followed by a rapid increase to 95% B within 1 min. The column was subsequently washed with 95% B and re-equilibrated for 10 min at a flow rate of 0.2 mL/min.

MS analysis was performed using an electrospray ionization (ESI)-based hybrid ion trap-Orbitrap mass spectrometer (Orbitrap Fusion™ Lumos™ Tribrid™ Mass Spectrometer). Full-scan spectra were acquired in the positive ion mode over an *m*/*z* range of 300–1800 at a resolution of 120,000.

Raw MS data were searched against the target protein database using PEAKS Studio 10.6. Database searches were conducted against the *Bos taurus* reference proteome (9913 entries) without any enzymatic cleavage specified, allowing up to 3 missed cleavages. The precursor mass tolerance was set to 20 ppm, and the fragment mass tolerance to 0.02 Da. Carbamidomethyl (C) was set as a fixed modification, whereas oxidation (M) and N-terminal acetyl (N-term) were set as variable modifications. The false discovery rate (FDR) was estimated based on the inherent decoy-fusion method. To ensure high-confidence peptide identification, a stringent dual-filtering strategy was applied. Specifically, only peptides with both a peptide-level FDR ≤ 1% and a peptide-spectrum match score of -10lgP ≥ 15 were retained for subsequent analyses.

The raw LC-MS/MS data files were further processed using MaxQuant v1.6.1.0. For peptide identification in fermented RW samples, a database containing all protein sequences from UniProtKB was employed. Semi-quantitative analyses were based on MS1 peak areas, which were normalized against the total ion current of each sample to adjust for experimental variance in loading and instrument sensitivity.

### 2.6 In silico analysis of the identified peptides

In silico approaches are widely used for the screening and analysis of bioactive peptides (Wei et al., 2023). The putative bioactive peptides were predicted using the PeptideRanker online tool (https://distilldeep.ucd.ie/PeptideRanker) and the BIOPEP-UWM online database (http://www.uwm.edu.pl/biochemia/index.php/pl/biopep). PeptideRanker was employed to assign a probability score (ranging from 0 to 1) indicating the likelihood of bioactivity, with a threshold value >0.5 defining putatively active sequences. Additionally, the identified peptides were searched against the BIOPEP-UWM database to annotate their potential biological activities based on exact match searches against validated sequences rather than homology-based inference.

### 2.7 Statistical analysis

Analysis of Variance (ANOVA) was employed to evaluate the temporal changes in pH and viable bacterial counts. This was followed by Tukey's Honestly Significant Difference (HSD) post hoc test for multiple comparisons. The semi-quantitative bioactive peptide data obtained from the whey samples were subjected to chemometric analysis using online software MetaboAnalyst 6.0 (Xia et al., 2015). Prior to constrained ordination, detrended correspondence analysis (DCA) was executed to assess gradient length. Because the gradient length of the first DCA axis was 1.78 (< 3.0, implying a linear response), redundancy analysis (RDA) was chosen over canonical correspondence analysis. Mantel tests and redundancy analysis (RDA) were performed using the Biomek Cloud platform (https://international.biocloud.net/). Associations between the putative bioactive peptides (peak areas normalized to the total ion current) and the relative abundances of the top 10 bacterial species were evaluated using Spearman's rank correlation analysis (*P* < 0.05). Data visualization was performed using GraphPad Prism (version 8.0.2).

## 3 Results and discussion

### 3.1 Changes in pH and viable counts during the spontaneous fermentation of RW

The proliferation and metabolic activity of LAB drove the marked and continuous acidification of RW during spontaneous fermentation. As a result, the pH showed a gradual decline and eventually stabilized at 3.49 by day 14. In parallel, the viable LAB count increased during the early stage and reached a plateau after 11 days. In contrast, viable yeasts were undetectable until day 14, at which point the population reached 6.02 log CFU/mL (Table 1).

**Table 1:** Changes in pH and viable microbial counts throughout RW fermentation.

Day	pH	LAB (CFU/mL)	Yeast (CFU/mL)
0	5.07 ± 0.04^a^	5.59 ± 0.16^a^	–
7	3.98 ± 0.01^b^	7.04 ± 0.06^b^	–
11	3.62 ± 0.01^c^	7.31 ± 0.05^c^	–
14	3.49 ± 0.01^d^	7.42 ± 0.06^c^	6.02 ± 0.17

At the onset of fermentation, LAB rapidly proliferated and became the predominant population. The metabolism of lactose by LAB caused a reduction in the pH. This acidic environment effectively inhibited the growth of competing bacteria and suppressed yeast activity. As fermentation proceeded, LAB entered the stationary phase, which attenuated acid production and weakened competitive inhibition. Moreover, the organic acids produced via LAB metabolism, together with the amino acids and peptides released from lysed LAB cells, subsequently facilitated the emergence of acid-tolerant yeasts. These yeasts became metabolically active and further contributed to the later stages of whey fermentation (Sieuwerts et al., 2008; Walsh et al., 2016).

Fermentation monitoring in this study was limited to pH and cell viability. Future work should systematically track key biochemical parameters (lactate, residual lactose, protease activity, and free amino acids) to fully elucidate how microbial metabolism drives peptide evolution during whey fermentation.

### 3.2 Microbial diversity in RW

In total, 432,873 reads of bacterial 16S rRNA genes were generated through high-throughput screening. After stringent quality control, 412,384 valid sequences were obtained, which accounted for 95.27% of the original reads. The Chao1 index analysis (Fig. 1A) showed that alpha diversity was significantly higher (*P* < 0.01) at the initial fermentation stage (F0d) than at the later stages. No significant differences were observed across the later fermentation stages (F7d, F11d, and F14d). Meanwhile, beta diversity analysis revealed distinct differences in bacterial diversity across the four fermentation stages (Fig. 1B). Firmicutes and Proteobacteria were the predominant taxa in all samples (Fig. 1C). As fermentation progressed, the abundance of Firmicutes increased initially, peaking during the middle stage (F7d: 80.89%). However, a subsequent decline was observed, followed by another rise during the final stage of fermentation. Proteobacteria followed the opposite trend, with their highest abundance recorded at F11d (44.38%).

**Fig. 1:**
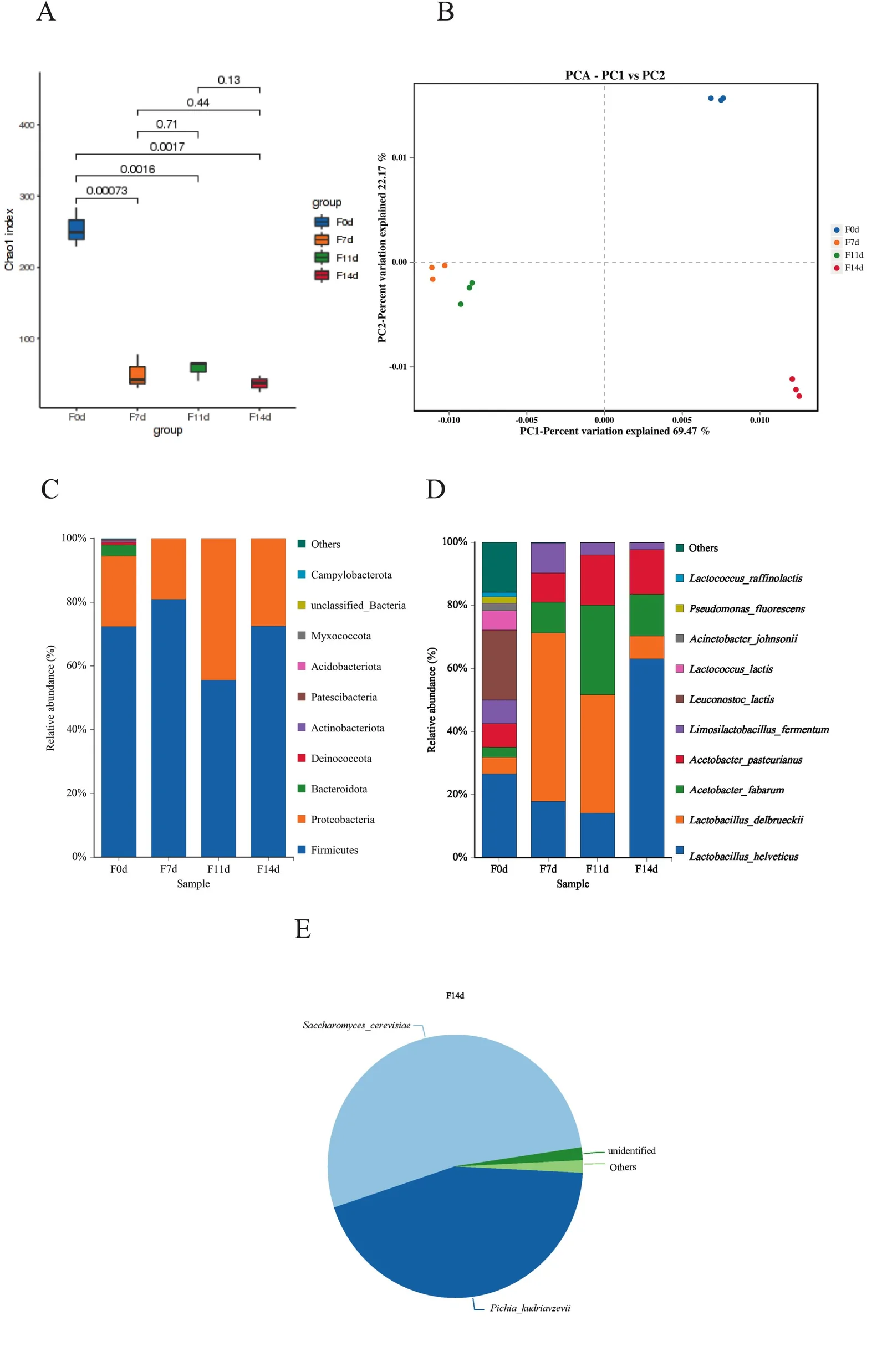
Microbial diversity during RW fermentation. (A) Bacterial α-diversity; (B) Bacterial β-diversity; (C) Bacterial community abundance at the phylum level; (D) Bacterial community abundance at the species level; (E) Fungal community abundance at the species level.

At the species level (Fig. 1D), the bacterial community appeared diverse during the initial stage of fermentation (F0d). *L. helveticus* (26.59%) and *Leuconostoc* (*Ln.*) *lactis* (22.12%) were found to be the most abundant species at this stage. As fermentation progressed, the microbial diversity decreased, but a few taxa became increasingly dominant. At F7d, *L. delbrueckii* became the predominant species (53.34%), whereas the abundance of L. *helveticus* declined (17.87%). Other species, including *Acetobacter fabarum, Acetobacter pasteurianus***,** and *Limosilactobacillus fermentum*, exhibited comparable abundance levels (approximately 9%). At F11d, the relative abundance of L. *delbrueckii, L. helveticus*, and L. *fermentum* decreased. However, the relative abundance of *A. fabarum* and *A. pasteurianus* increased during this stage. By F14d, *L. helveticus* emerged as the sole dominant species (62.99%), whereas the abundance of *A. fabarum* and L. *delbrueckii* decreased markedly. These findings were consistent with previous studies, which reported that L. *delbrueckii* and L. *helveticus* were commonly found in the whey obtained from cheese production (Ercolini et al., 2008).

Interestingly, during the first three stages of fermentation, fungi were undetectable in the RW samples. However, relatively high abundances of *Saccharomyces cerevisiae* and *Pichia kudriavzevii* were detected in the samples after 14 days of fermentation. These findings necessitate a consideration of methodological sensitivity and detection limits. The fungal DNA titer in the early fermentation samples was below the detection limit required for successful amplicon library construction. As a result, fungal sequencing data could not be obtained before day 14. The emergence of fungi on day 14 marked a critical ecological tipping point, driven by bacterial–fungal interactions, which allowed the fungi to rapidly proliferate and surpass the methodological detection threshold. Finally, linear discriminant analysis (LDA) effect size (LEfSe) analysis was performed to identify the bacterial taxa that contributed to community differences across different fermentation stages (Fig. 2). A total of 30 taxa (LDA score > 4) were identified as key discriminative biomarkers. The early stage (F0d) was characterized by the enrichment of *L. lactis*, *Ln. lactis*, and *Acinetobacter johnsonii*. By the middle stage of fermentation (F7d), L. *delbrueckii* and *L. fermentum* emerged as the predominant bacterial species. *A. fabarum* and *A. pasteurianus* were significantly enriched at F11d, whereas L. *helveticus* characterized the final stage (F14d).

**Fig. 2:**
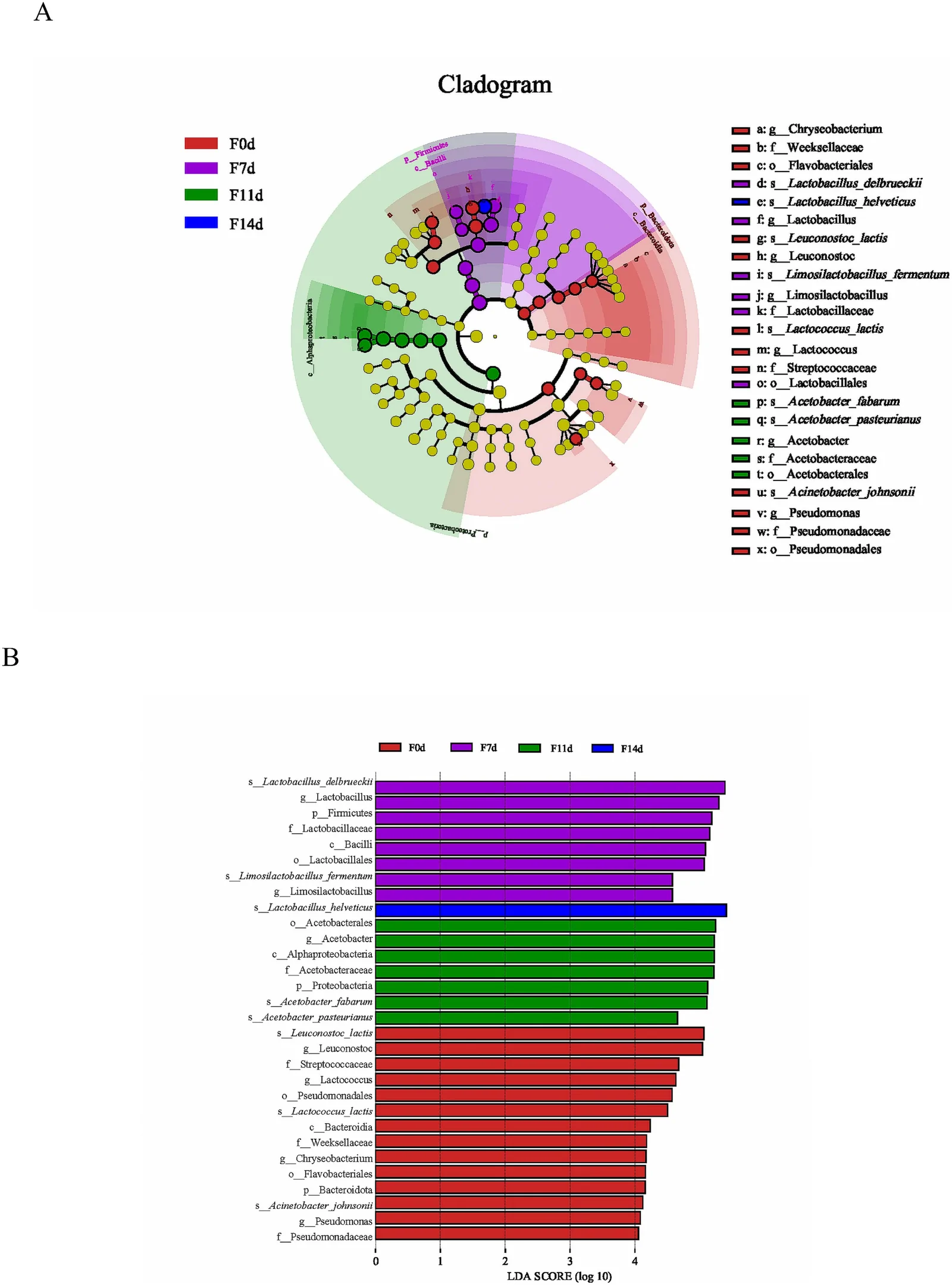
Linear discriminant analysis (LDA) combined with effect size (LEfSe) of the bacterial composition of RW. (A) Phylogenetic distribution of differentially abundant taxa; (B) Histogram of LDA scores (threshold >4.0) showing the taxa with significant abundance differences between the groups.

### 3.3 Peptidomic analysis of RW at different fermentation stages

In total, 8532 peptides were identified across the four fermentation stages through LC-MS/MS analysis. To visualize the differences in peptide variety during fermentation, a Venn diagram was created (Fig. 3A). The number of detected peptides gradually decreased as fermentation was prolonged (4724 at F0d, 2691 at F7d, 1956 at F11d, and 1667 at F14d). Notably, 516 peptides were detected at all four stages, whereas 3454, 906, 424, and 415 peptides were specific to F0d, F7d, F11d, and F14d, respectively. In the F0d sample, peptides with molecular weights ranging from 1000 to 1500 Da were highly abundant. By contrast, peptides with molecular weights below 1000 Da were abundant in the other samples (F7d, F11d, and F14d) (Fig. 3B).

**Fig. 3:**
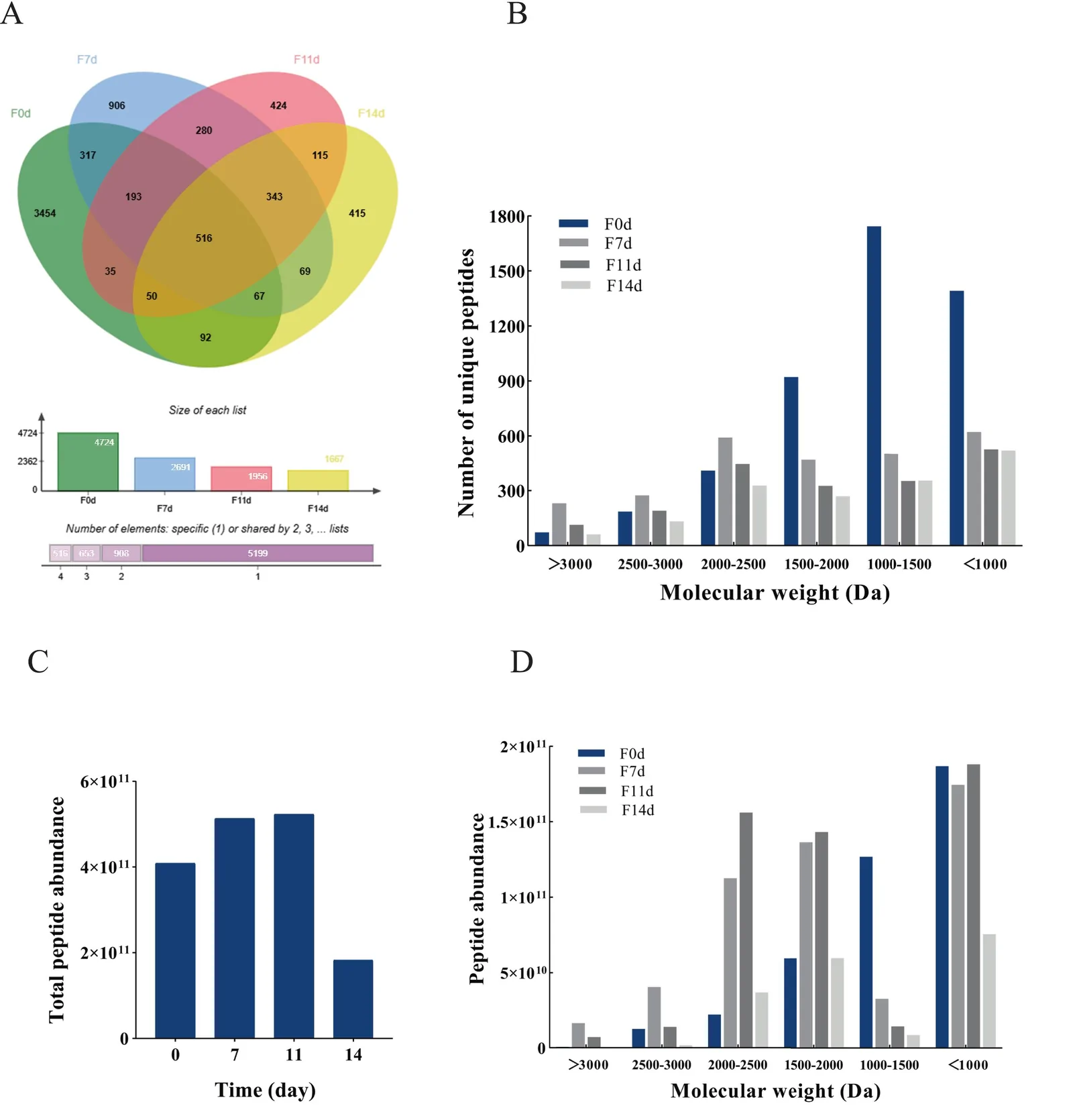
Peptidomic characterization during RW fermentation. (A) Venn diagram of the identified peptides; (B) Distribution of unique peptides according to their molecular weight (in Da); (C) Total peptide abundance; (D) Distribution of peptide abundance based on molecular weight (in Da).

Different microbial strains exhibit distinct proteolytic profiles. Therefore, higher microbial diversity is often associated with a broader range of enzymatic degradation pathways, contributing to greater diversity of peptides (Kieliszek et al., 2021). In this study, the highest peptide diversity was observed at F0d; this could be attributed to the presence of various microbial enzymes, which facilitated extensive protein hydrolysis. As fermentation progressed, the dominance of a few species with narrow enzymatic specificity led to the further degradation of peptides into simpler fragments, causing a reduction in peptide diversity (Savijoki et al., 2006).

Peptide abundance analysis revealed a marked variation across different fermentation stages. Overall, the peptide abundance increased during the early and middle stages of fermentation, peaking on day 11 and subsequently declining to its lowest levels by day 14 (Fig. 3C). Peptides with molecular weight below 1000 Da consistently exhibited a higher abundance across all fermentation stages (Fig. 3D).

Microbial growth and metabolism generate enzymes that facilitate the hydrolysis of proteins into lower-molecular-weight peptides. Additionally, they enable the further breakdown of peptides into smaller fragments and amino acids, ultimately contributing to a reduction in overall peptide abundance (Zou et al., 2024). In this study, the high fungal abundance observed at F14d may have also facilitated peptide degradation into smaller molecules, such as amino acids. A previous study by Ahmed et al. reported that peptides with molecular weights below 3000 Da exhibit high bioavailability and significant bioactivity (Ahmed et al., 2015). Given that the majority of peptides identified in this study fell below this molecular weight threshold, the results suggested that fermented RW represents a promising source of bioactive peptides.

RW contains a diverse range of proteins that can be hydrolyzed by microbial enzymes into a wide variety of peptides. Casein accounts for approximately 80% of the total protein in milk and has an isoelectric point of pH 4.6. The relatively high casein content of RW can be attributed to incomplete casein coagulation during Rushan production, as the curdling pH is typically maintained above pH 5.

Of the 15 most abundant RW peptide sources identified in this study, six were milk proteins and nine were milk protein precursors (Fig. 4A). Peptide profiling was conducted to visualize peptide characteristics such as amino acid sequence, start position, length, and protein origin (Amorim et al., 2019). Accordingly, peptide maps (Fig. 4B) were generated for the top four most abundant proteins detected across different fermentation stages: CASB (β-casein), CASA1 (αS1-casein), CASA2 (αS2-casein), and CASK (κ-casein).

**Fig. 4:**
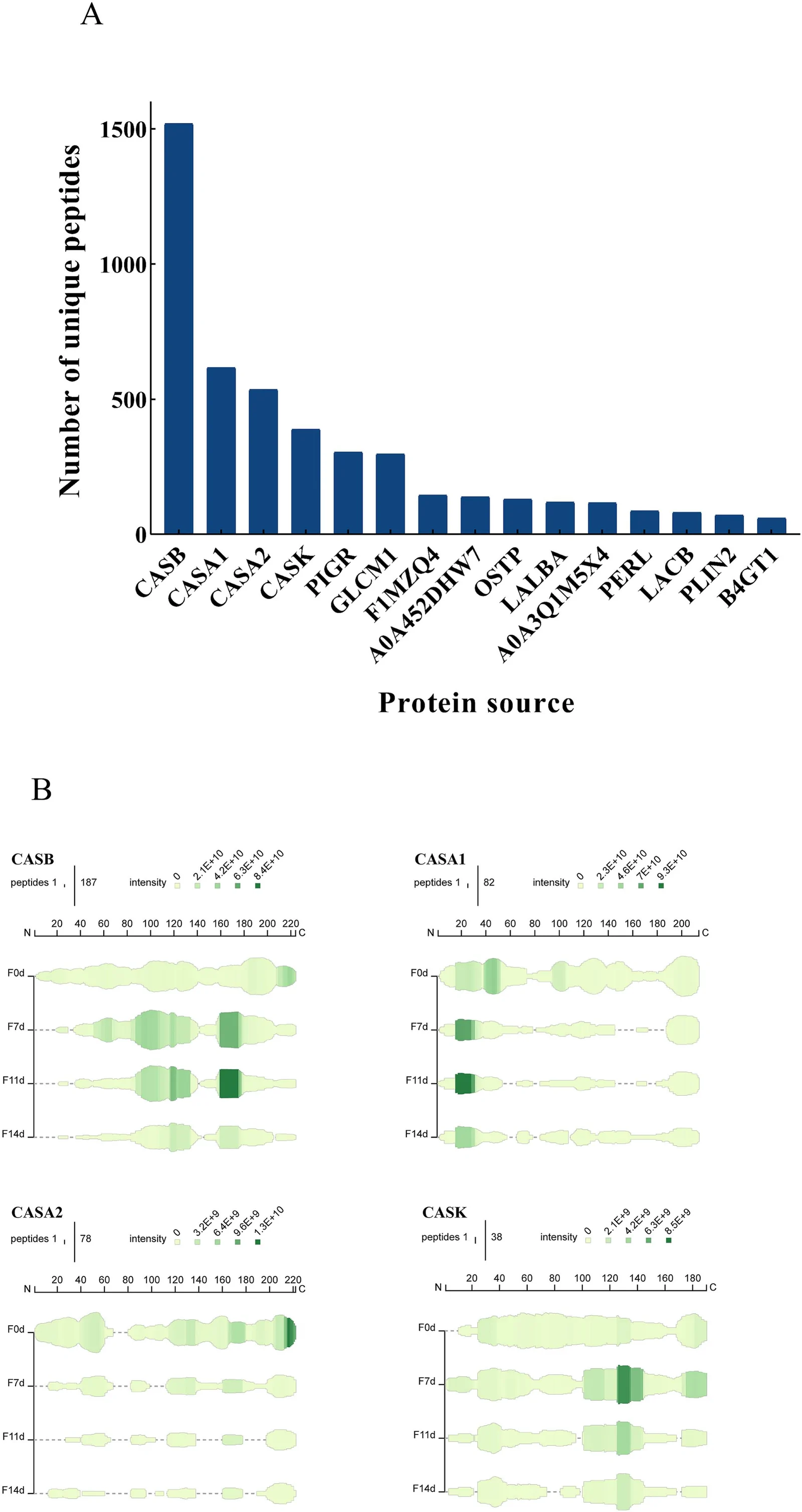

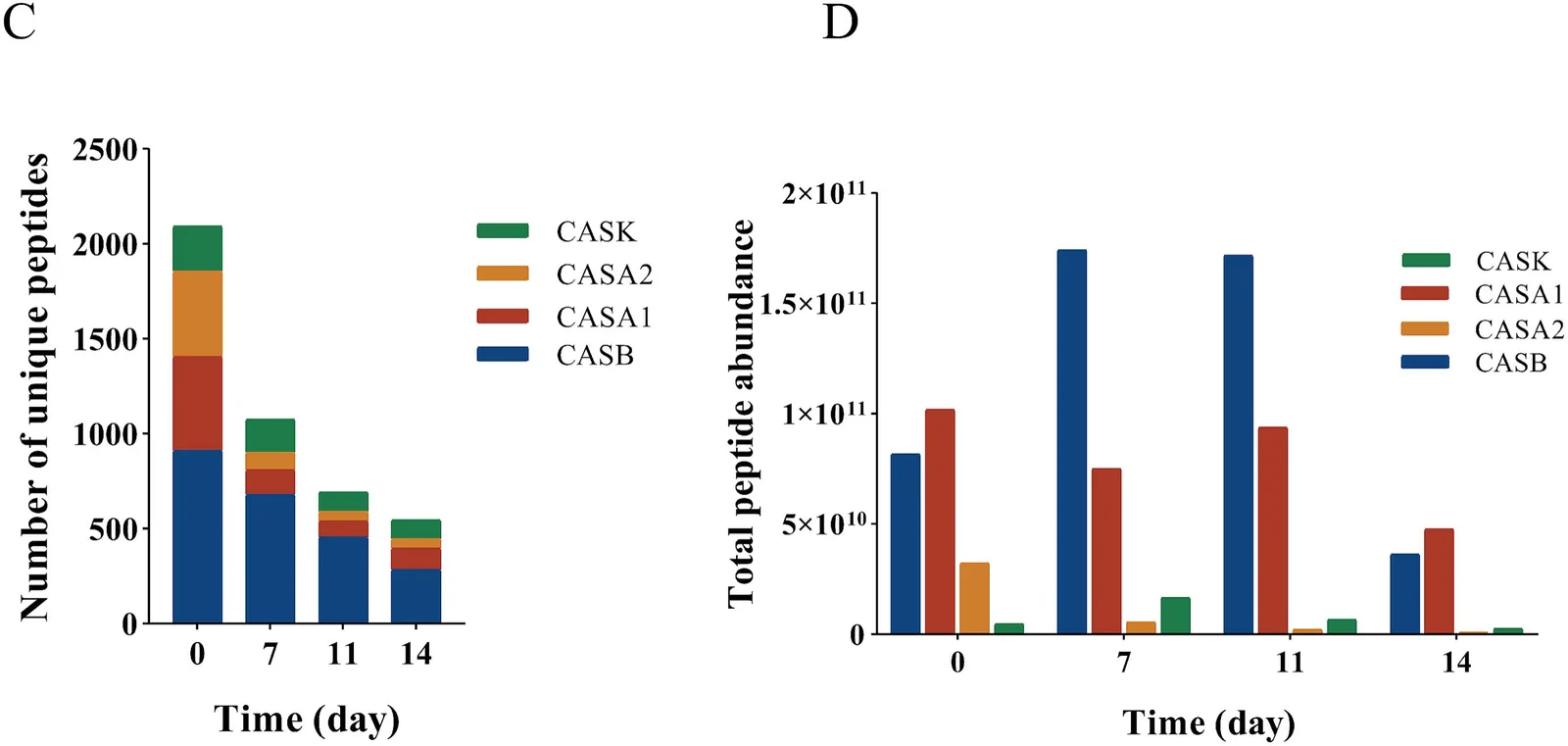
(A) Number of peptides from the top 15 RW protein sources (with the highest peptide quantity); (B) Maps of peptides derived from the major RW proteins across different fermentation stages. The height of each bar indicates the number of peptide segments, while the color intensity corresponds to the mass spectrometry response value of each peptide; (C) Number of peptides derived from major RW proteins at each fermentation stage; (D) Abundance of peptides originating from major RW proteins throughout fermentation.

The peptides derived from some major proteins shared their positions on the peptide map across specific fermentation stages. Notably, the peptide maps of samples fermented for 7 (F7d) and 11 days (F11d) showed greater similarity (Fig. 4B); however, the peptide abundance at these stages was different. In contrast, the F0d sample displayed a distinct peptide profile, with relatively abundant peptide segments. The changes in peptide profiles observed throughout fermentation corresponded to the shifts in bacterial composition. This was likely because the variations in microbial communities during fermentation altered the types and activities of enzymes produced. Since the bacterial communities at F7d and F11d were relatively similar, the resulting peptide profiles exhibited only minor differences.

For CASB and CASA1, the changes in peptide profiles were relatively similar. The F11d sample contained a relatively high abundance of both proteins. A prominent AA160–176 fragment was detected in CASB, and a rich AA15–28 fragment was detected in CASA1. Notably, the abundance of CASA2 peptide segments was higher in the F0d sample than in the other samples (F7d, F11d, and F14d). This suggested that microbial fermentation did not enhance the hydrolysis of CASA2. This could be attributed to the inertness of the microbial enzymes present in RW toward CASA2. In addition, an AA125–135 fragment derived from CASK was found to be specifically enriched in the F7d sample.

LAB possess diverse protein hydrolysis systems, including cell-envelope proteinase (CEP) (Savijoki et al., 2006). These enzymatic systems facilitate the breakdown of proteins and the release of numerous peptides with high bioavailability and potential biological activity. As the microbial community stabilizes during fermentation, the overall peptide profile can exhibit relatively limited variation (Zou et al., 2024).

In this study, the analysis of peptide sources throughout the fermentation process (Fig. 4C and D) indicated that peptides derived from CASB were both the most numerous and most abundant. This predominance was closely linked to the structural properties of casein, particularly CASA1, CASA1, and CASB, which are present in high levels and possess flexible, open molecular conformations. Such features enhance their susceptibility to proteolytic enzymes, thereby promoting cleavage and the generation of bioactive peptides (Wei et al., 2022). Additionally, CASB is reported to be a preferred substrate for the CEP produced by LAB, explaining the predominance of peptides derived from its hydrolysis in RW (Martini et al., 2024).

### 3.4 Analysis of putative bioactive peptides in RW

In MS-based peptide identification, higher ion scores indicate a greater reliability and accuracy for peptide assignment (Chepanoske et al., 2005). Accordingly, peptides with scores exceeding 45 (-10lgP > 45) were selected for further analysis in this study. A total of 107 non-modified peptides were subjected to bioactivity prediction using the PeptideRanker tool, with peptides scoring above 0.5 being considered putatively bioactive (Table S1). In total, 39 peptides that met this criterion were screened.

As fermentation progressed, the proportion of peptides with bioactivity scores >0.5 increased gradually (Fig. 5A). However, the absolute abundance of these putative bioactive peptides peaked on day 7 before declining to their lowest levels on day 14. The temporal divergence between the peak of total peptides (day 11, Fig. 3C) and putative bioactive peptides (day 7, Fig. 5B) reflected the sequential nature of microbial proteolysis. Initially, the microbial proteinases rapidly released intermediate-sized bioactive peptides. As fermentation progressed beyond day 7, these peptides were further degraded into smaller, inactive fragments, ultimately driving the delayed peak of total peptide abundance (day 11). This dynamic highlights the importance of optimizing the fermentation time to maximize the yield of specific bioactive peptides.

**Fig. 5:**
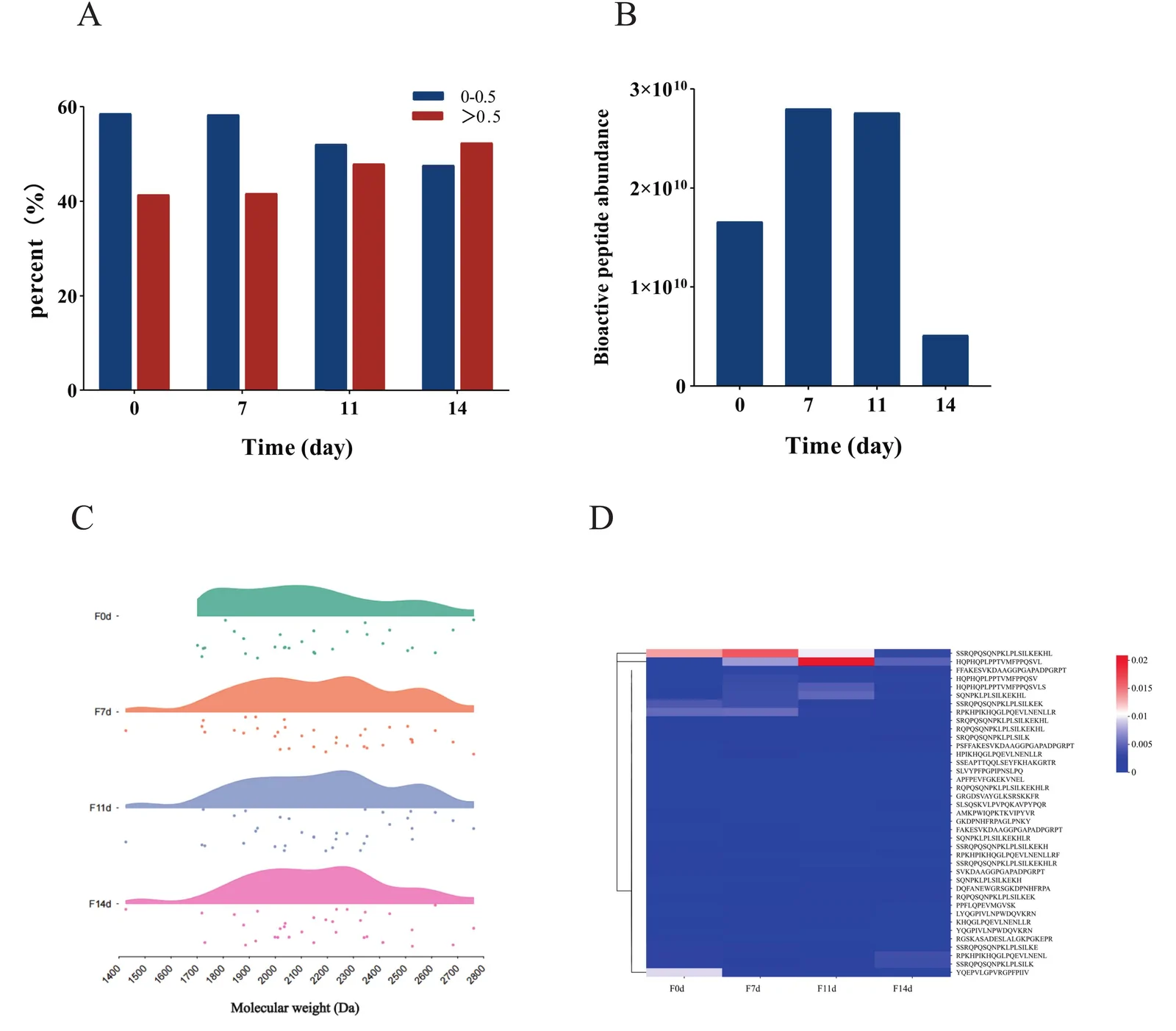
Analysis of bioactive peptides in RW during fermentation. (A) Proportion of peptides with different bioactivity scores; (B) Abundance of putative bioactive peptides; (C) Molecular weight distribution of putative bioactive peptides; (D) Peak areas of representative bioactive peptides.

An analysis of molecular weight distribution demonstrated that across all fermentation stages, the putative bioactive peptides in RW largely had a molecular weight of 1000–3000 Da (Fig. 5C). This distribution was consistent with previous findings that peptides within the range of 1000–3000 Da exhibit enhanced biological target binding and improved physiological functions (Shi et al., 2021). Similarly, Wei et al. reported that Angiotensin-Converting Enzyme (ACE) inhibitory and antioxidant peptides derived from fermented dairy products were mainly distributed within the molecular weight range of 1000–3000 Da (Wei et al., 2022). Fig. 5D illustrates the changes in the abundance of representative putative bioactive peptides during fermentation. Collectively, these results demonstrated that the bioactive potential of RW may be effectively modulated through microbial fermentation.

Using the BIOPEP-UWM database, a total of 19 putative bioactive peptides were identified (Table 2). These peptides were theoretically capable of functional effects such as immunomodulatory, antioxidant, antibacterial, and Calcium/Calmodulin-Dependent Phosphodiesterase (CaMPDE) inhibitory activities. Peptides sharing identical sequence motifs were predicted to possess the same potential bioactive functions. In the present study, peptides containing the sequence RPKHPIKHQGLPQE were consistently associated with potential immunomodulatory, antioxidant, and antibacterial activities. The peptides derived from CASA1 showed a high abundance and diverse bioactivities, warranting further in-depth examination. Overall, the findings suggested that fermented RW has the potential to serve as a source of functional bioactive peptides. However, in silico predictions have certain limitations that must be considered. The multifunctional activities predicted through these analyses require validation through subsequent in vitro functional experiments. In addition, from an industrial scalability perspective, the endogenous peptidomic profiles obtained in this study only provide valuable molecular targets for the functional food and dairy industries. While our analytical extraction methods (e.g., LC-MS/MS sample preparation) are not intended for large-scale production, the identified high-abundance and putative bioactive peptides could be effectively enriched at an industrial scale using well-established, non-destructive technologies, such as advanced membrane filtration and preparative chromatography. Given that sequence identification and relative quantification were the main goals of this work, reduction and alkylation were implemented during peptide extraction. However, these steps may alter disulfide-bonded and cysteine-containing peptides, and thus the identified peptidome may not fully represent native peptide structures. Future work should include non-reducing and non-alkylating extraction conditions for comparison.

**Table 2:** Properties of the putative bioactive peptides detected in RW.

NO.	Peptide	Protein Accession	Activity	Length	Mass	-10lgP
1	RPKHPIKHQGLPQE	CASA1	Antibacterial, immunomodulating, antiviral	14	1663.927	49
2	RPKHPIKHQGLPQEVL	CASA1	Antibacterial, immunomodulating, antiviral	16	1876.0795	46.73
3	KHQGLPQEVLNENLLR	CASA1	Antibacterial, immunomodulating, antiviral	16	1887.0326	45.52
4	RPKHPIKHQGLPQEVLNE	CASA1	Antibacterial, immunomodulating, antiviral	18	2119.165	49.37
5	HPIKHQGLPQEVLNENLLR	CASA1	Antibacterial, immunomodulating, antiviral	19	2234.2283	49.84
6	RPKHPIKHQGLPQEVLNEN	CASA1	Antibacterial, immunomodulating, antiviral	19	2233.208	54.42
7	RPKHPIKHQGLPQEVLNENL	CASA1	Antibacterial, immunomodulating, antiviral	20	2346.292	50.37
8	RPKHPIKHQGLPQEVLNENLL	CASA1	Antibacterial, immunomodulating, antiviral	21	2459.376	52.64
9	RPKHPIKHQGLPQEVLNENLLR	CASA1	Antibacterial, immunomodulating, antiviral	22	2615.4771	54.63
10	RPKHPIKHQGLPQEVLNENLLRF	CASA1	Antibacterial, immunomodulating, antiviral	23	2762.5457	55.04
11	HVSSSEESIISQETYK	CASA2	Immunomodulating	16	1822.8584	48.29
12	YQEPVLGPVRGPFPIIV	CASB	Immunomodulating	17	1880.0559	48.12
13	MEHVSSSEESIISQETYK	CASA2	Immunomodulating	18	2082.9417	48.87
14	SKVLPVPQKAVPYPQ	CASB	Antioxidative	15	1649.9504	45.81
15	SQSKVLPVPQKAVPYPQ	CASB	Antioxidative	17	1865.041	45.72
16	DDTGTPITKIELVPSH	NPT2B	Antioxidative	16	1721.8835	45.92
17	AMKPWIQPKTKVIPYVR	CASA2	CaMPDE-inhibiting	17	2054.1863	50.64
18	APKHKEMPFPKYPVEPFTE	CASB	Stimulating	19	2271.1396	47.88
19	VADYGKDLVEKAKGSELQTQAK	APOA2	Antibacterial	22	2377.2488	45.82

### 3.5 Correlation between bacterial composition and peptide abundance

Peptide generation in RW is mediated by microbial activity, mainly through the action of dominant LAB. The CEP produced by LAB initially hydrolyzes milk proteins into oligopeptides (2–20 amino acids). These oligopeptides are subsequently transported into cells via specific peptide transport systems. Thereafter, these oligopeptides are broken down into smaller peptides or free amino acids through the coordinated action of various intracellular peptidases. Because different bacterial strains exhibit distinct proteolytic specificity and enzymatic profiles, the peptide composition of fermented whey is largely shaped by dynamic changes in its microbial community structure (Guo et al., 2016). Throughout fermentation, multiple enzyme systems secreted by LAB, including endopeptidases, dipeptidases, and aminopeptidases, collectively contribute to the degradation of RW proteins (Shi et al., 2021; Solieri et al., 2022).

In this study, the Mantel test (Fig. 6A) revealed a highly significant correlation (*P* < 0.01) between the bacterial community structure of RW and the abundance of total peptides as well as peptides derived from major RW proteins. These results indicated that microbial composition may be strongly associated with the abundance of peptides, especially those derived from CASB (*r* ≥ 0.4).

**Fig. 6:**
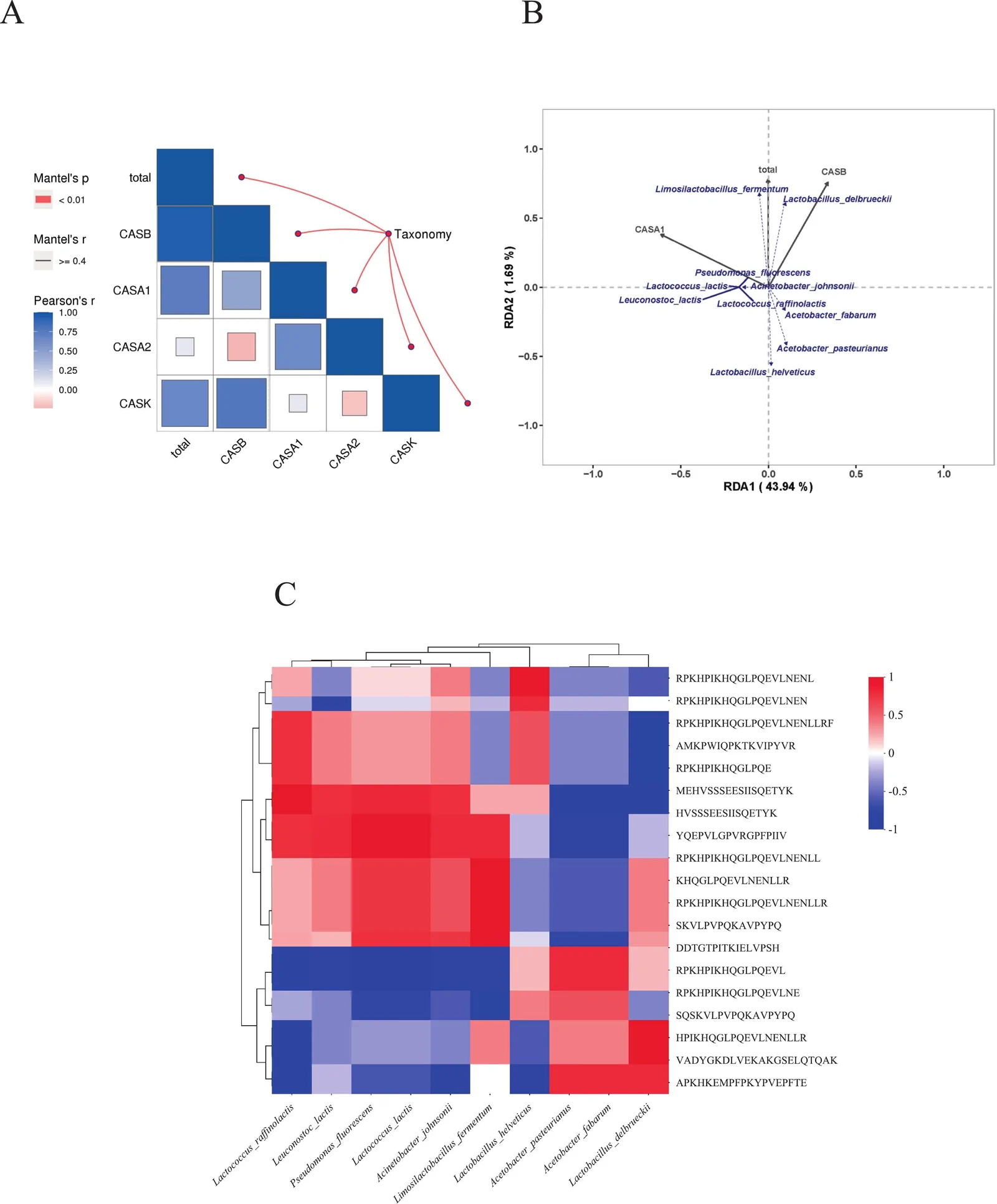
Correlation between peptide and bacterial abundance in RW. (A) Mantel test of the relationship between bacterial communities and protein sources; (B) RDA of the relationship between protein sources and the top 10 most abundant bacterial species; (C) Spearman correlation analysis of the relationship between putative bioactive peptides and the top 10 most abundant bacterial species.

RDA further clarified the relationships between the top 10 most abundant bacterial species and protein sources (Fig. 6B). L. *fermentum* and *L. delbrueckii* were positively correlated with the abundance of total peptides, as well as peptides derived from CASB and CASA1. In contrast, *Lactococcus raffinolactis*, *A. johnsonii*, *Pseudomonas fluorescens*, *L. lactis*, and *Ln. lactis* showed a positive correlation with total peptides and CASA1-derived peptides, but they exhibited a negative correlation with CASB-derived peptides. Additionally, *A. fabarum*, *A. pasteurianus*, and L. *helveticus* were negatively correlated with the abundance of total peptides as well as CASB- and CASA1-derived peptides.

The proteolytic activities of LAB vary substantially among species. Previous studies have reported that, among dairy-associated LAB, *Lactobacillus ylae*, *Lactobacillus delbrueckii* subsp. *lactis*, *L. helveticus*, and L. *raffinolactis* exhibit strong proteolytic activity, whereas *Lactobacillus plantarum* demonstrates relatively weak activity (Lim et al., 2019; Toe et al., 2019).

To further assess the relationship between specific bacterial species and the 19 putative bioactive peptides, Spearman correlation analysis was performed using microbial composition and peptide abundance data (Fig. 6C). The results showed that the correlation patterns between different bacterial species and putative bioactive peptides varied considerably. *L. helveticus*, which exhibited a relatively high relative abundance throughout fermentation, showed positive correlations with 8 putative bioactive peptides and negative correlations with 6. Similarly, *L. delbrueckii* showed positive correlations with 8 peptides and negative correlations with 8. These results suggested a strong statistical association between the presence of specific bacterial species and some putative bioactive peptides.

It is important to note that these findings only reflected the associations between bacterial taxa and peptide profiles. However, as functional enzyme activities and related gene expression profiles were not analyzed, no direct causality could be established. Nevertheless, we speculate that differences in bacterial proteolytic systems may contribute to variations in peptide generation. Conversely, the peptides generated may also influence the assembly of microbial communities, thereby modulating microbial diversity (Kieliszek et al., 2021). The specific proteolytic roles of individual bacterial species require validation through fermentation with isolated strains and transcriptomic analysis.

## 4 Conclusion

Overall, this study systematically characterized the dynamics of microbial communities and peptide profiles during the spontaneous fermentation of RW. Although a conserved core bacterial community was maintained in RW during fermentation, its structure was considerably altered, with L. *helveticus* and L. *delbrueckii* becoming dominant in the later stages of fermentation. In contrast, the fungal community was only detectable on day 14.

Peptidomic analysis revealed that peptide diversity declined over the fermentation period, whereas the overall peptide abundance and potential bioactivity peaked at the mid-fermentation stage. Most of the identified peptides were derived from CASB, with CASA1 being the second most prevalent source. Bioinformatic analysis identified 39 peptides with potential bioactivity, of which 19 were predicted to be putatively functional. Correlation analysis revealed that the bacterial community structure was significantly correlated with the putative bioactive peptides in naturally fermented RW, providing a theoretical basis for exploiting indigenous LAB strains to promote the production of bioactive peptides. However, the current findings were derived from a single industrial batch of RW. Given the inherent variability of spontaneous fermentation, additional studies incorporating multiple independent batches of whey across different seasons and production sites are warranted to confirm the universal nature of these microbe–peptide interactions. Nevertheless, the findings demonstrate that fermented RW serves as a rich source of functional peptides, establishing a scientific foundation for the targeted enrichment of bioactive sequences through controlled microbial fermentation. Ultimately, this work provides a sustainable strategy for converting this underutilized dairy byproduct into high-value functional food ingredients. Future research should focus on isolating key functional microbial strains and validating the bioactivity of RW-derived peptides.

The following are the supplementary data related to this article.

Supplementary Table S1 Putatively bioactive peptides identified in RW.

## CRediT authorship contribution statement

**Rong Tang:** Writing – original draft, Methodology, Formal analysis, Data curation, Conceptualization. **Huimin Li:** Project administration, Funding acquisition. **Faguang Yang:** Resources, Investigation. **Xing Wan:** Writing – review & editing, Validation. **Xingying Dai:** Methodology, Investigation. **Junfei Chen:** Software, Investigation. **Chenrui Zhang:** Resources, Methodology. **Ling Zou:** Writing – review & editing. **Qiao Shi:** Writing – original draft, Funding acquisition.

## Declaration of competing interest

The authors declare that they have no known competing financial interests or personal relationships that could have appeared to influence the work reported in this paper.

## Data Availability

The mass spectrometry proteomics data have been deposited to the ProteomeXchange Consortium via the PRIDE partner repository with the dataset identifier PXD082962. Other data are available from the corresponding author upon reasonable request.
